# The inhibition of the mammalian DNA methyltransferase 3a (Dnmt3a) by dietary black tea and coffee polyphenols

**DOI:** 10.1186/1471-2091-12-16

**Published:** 2011-04-21

**Authors:** Arumugam Rajavelu, Zumrad Tulyasheva, Rakesh Jaiswal, Albert Jeltsch, Nikolai Kuhnert

**Affiliations:** 1 Chemistry, Jacobs University Bremen, Campus Ring 1, 28759 Bremen, Germany; 2 Biochemistry, Jacobs University Bremen, Campus Ring 1, 28759 Bremen, Germany; 3 MoLife program, Jacobs University Bremen, Campus Ring 1, 28759 Bremen, Germany

## Abstract

**Background:**

Black tea is, second only to water, the most consumed beverage globally. Previously, the inhibition of DNA methyltransferase 1 was shown by dietary polyphenols and epi-gallocatechin gallate (EGCG), the main polyphenolic constituent of green tea, and 5-caffeoyl quinic acid, the main phenolic constituent of the green coffee bean.

**Results:**

We studied the inhibition of DNA methyltransferase 3a by a series of dietary polyphenols from black tea such as theaflavins and thearubigins and chlorogenic acid derivatives from coffee. For theaflavin 3,3 digallate and thearubigins IC_50 _values in the lower micro molar range were observed, which when compared to pharmacokinetic data available, suggest an effect of physiological relevance.

**Conclusions:**

Since Dnnmt3a has been associated with development, cancer and brain function, these data suggest a biochemical mechanism for the beneficial health effect of black tea and coffee and a possible molecular mechanism for the improvement of brain performance and mental health by dietary polyphenols.

## Background

Black tea is, second only to water, the most consumed beverage globally with an average per capita consumption of around 550 ml per day. The annual production of tea leaves reached a record high in 2008 with a global harvest of 3.75. Mt [1]. Production of dried tea comprises 20% green, 2% oolong and the remainder black. Following black tea, coffee is the third most consumed beverage globally with an annual production of 9.7 Mt and a daily consumption of around 300 ml (data from http://www.fas.usda.gov/, obtained 1st March 2011). Strong epidemiological evidence has repeatedly linked the consumption both black tea [2] and coffee [3,4] to a variety of beneficial health effects, among them is the prevention of multifactorial diseases including cancer, cardiovascular disease and neurological disorders as well as a series of psychoactive responses improving alertness, mood and general mental performance [5-8]. Recently, Unilever made an application for a health claim, in which the black tea beverage should supposedly improve mental alertness and focus, based on studies by Nurk et al. with the activities of the two compounds caffeine and L-theanine as the proposed rationale [9]. While epidemiological studies link two causally unrelated events, e. g. a beneficial health effect with the consumption of a certain diet, with a certain statistical probability, the molecular causes of these epidemiological observations are rarely known. In order to rationalize epidemiological observations, a biological target must be identified that is mechanistically linked to the beneficial health effect reported, as well as the specific molecules contained in the diet that interact with the biological target in question at dietary and physiologically relevant concentrations. The search for such matching pairs of biological targets and dietary compound must be considered an exercise of fishing in the dark, however, where enzymes known to be intimately involved in the area in question need to be systematically screened against secondary metabolites known to be produced by the dietary plant in question.

Prompted by reports of Fang and co-workers, who have recently reported the inhibition of DNA methyltransferase 1 (Dnmt1) by a series of dietary polyphenols [10] and work by Lee and co-workers on the inhibition of the same enzyme investigating most notably epi-gallocatechin gallate (EGCG) [11] (the main polyphenolic constituent of green tea) and 5-caffeoyl quinic acid [12] (the main phenolic constituent of the green coffee bean), and Nandakumar, showing the reduction of cellular DNA methylation after admission of (-)-epigallocatechin-3-gallate [13], we decided to screen the interaction of a series of black tea and coffee polyphenols against DNA methyltransferase 3a, another important member of this family of enzymes.

DNA methyltransferases catalyzes methylation of DNA at cytosine residues and play an important role in epigenetic regulation of gene expression, X-chromosome inactivation, genomic imprinting, and development cellular aging and cell differentiation [14,15]. In mammals, DNA methylation is catalyzed mainly by three DNA methyltransferases [15,16]: Dnmt1, Dnmt3a, and Dnmt3b. Dnmt1 has a high preference for hemimethylated DNA and is essential for maintaining the methylation patterns during each round of DNA replication. On the other hand, Dnmt3a and Dnmt3b modify both unmethylated and hemimethylated DNA and are responsible for *de novo *methylation during early development. Errors in DNA methylation contribute to both the initiation and the progression of various cancers [17,18]. In addition, aberrant or missing DNA methylation causes many kinds of diseases which include defects in embryonic development or brain development and neurological defects which are also associated with behavioral changes [19]. Hypermethylation of genes is one of important process in cancer development, typically resulting in the repression of tumor suppressor genes. Preventing the hypermethylation of promoter genes by selective inhibition of methyltransferases could pave a way for cancer treatment [20-22]. Importantly it has been shown that upon use of methyltransferase inhibitors it was possible to reactivate gene silenced by promoter methylation in cancers and thus modulate gene expression. Several efforts are directed at developing small molecules that target DNA methyltransferases and other elements of the machinery, as the proteins that bind to methylated CpG; some are in clinical trials [20-22].

Another important issue of DNA methylation is its function in brain development. Levenson and coworkers showed that Dnmt1 is involved in the formation of hippocampus-dependent long term memory [23]. They found that the promoters for reelin and brain-derived neurotrophic factor (genes implicated in the induction of synaptic plasticity in the adult hippocampus) exhibit rapid and dramatic changes in cytosine methylation when Dnmt1 activity was inhibited. Moreover, DNA methyltransferase inhibitors like 5-aza-2-deoxycytidine blocked the induction of long term potentiation at Schaffer collateral synapses. Furthermore, Dnmt3a-dependent DNA methylation has been reported to influence transcription of neurogenic genes [24]. Additional studies showed that Dnmt1 and Dnmt3a regulate synaptic function in adult forebrain neurons [25] and Dnmt3a affects plasticity of neurons [26].

Changes in the DNA methylation pattern of regions in the hippocampus are associated with behavioral changes in rat [27]. In addition, Dnmt3a has been recently shown to affect the emotional behaviour [26]. Thus, DNA methylation which is already known to be involved in setting up cellular memory is also involved in brain function. The combination of studies in cell lines and in animal models, coupled with data obtained from post-mortem human material provides compelling evidence that aberrant methylation may contribute to psychiatric diseases like schizophrenia and psychosis [28]. Strong epidemiological evidence suggests that particularly for black tea and green tea there is an inverse relation between intake and significant beneficial effects on patients suffering from psychological disorders [2,5-8]. Currently, no accepted rationale on the molecular level exists that can account for these epidemiological findings. Dnmts are a possible biological target for tea dietary polyphenols suggesting a molecular based rationale for the observed biological activities.

## Results

### Expression and purification of Dnmt3a-C

**Figure 1:**
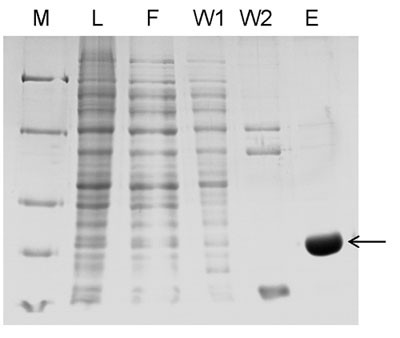
**Purification of Dnmt3a catalytic domain**. Fig. 1 Purified Dnmt3a-C separated on 12% SDS-PAGE gel and stained with colloidal Coomassie Blue. (M, Size marker for 116, 66.2, 45, 35 and 25 kDa; L, crude lysate; F, Flow through; W1, Wash1; W2, Wash2; E, Elution). The purified Dnmt3a-C protein runs at an apparent size of 36 kDa (highlighted with an arrow).

The catalytic domain of Dnmt3a was expressed and purified following an established protocol [29,30]. The purified protein by Ni-NTA affinity chromatography was >90% homogenous as judged from SDS-PAGE stained with colloidal Coomassie Blue (Figure 1).

### Selection and purification of black tea and coffee polyphenols

Black tea is produced from the young green shoots of the tea plant (*Camellia sinensis*), which are converted to black tea by fermentation [31]. There are two major processes, the 'orthodox' and the 'cut-tear-curl'. In both, the objective is to achieve efficient disruption of the cellular substructure bringing phenolic compounds present in the green tea leaf, mainly flavan-3-ols otherwise known as catechins, into contact with polyphenol oxidases and activating many other enzymes. The catechin substrates are oxidized and extensively transformed into novel dimeric, oligomeric and polymeric compounds. The chemical composition of black tea brew can be divided into (i) a series of well characterized small molecules including alkaloids (e.g. theobromine and caffeine), carbohydrates and amino acids (including theanine), and a series of glycosylated flavonoids and dimers of catechins including most notably theaflavins together accounting for 30-40% of the dry mass of a typical black tea infusion, and (ii) the heterogeneous and poorly characterized polyphenolic fermentation products accounting for the remaining 60-70% [31]. This material was originally referred to as oxytheotannin and later renamed by Roberts as thearubigins [32].

**Figure 2:**
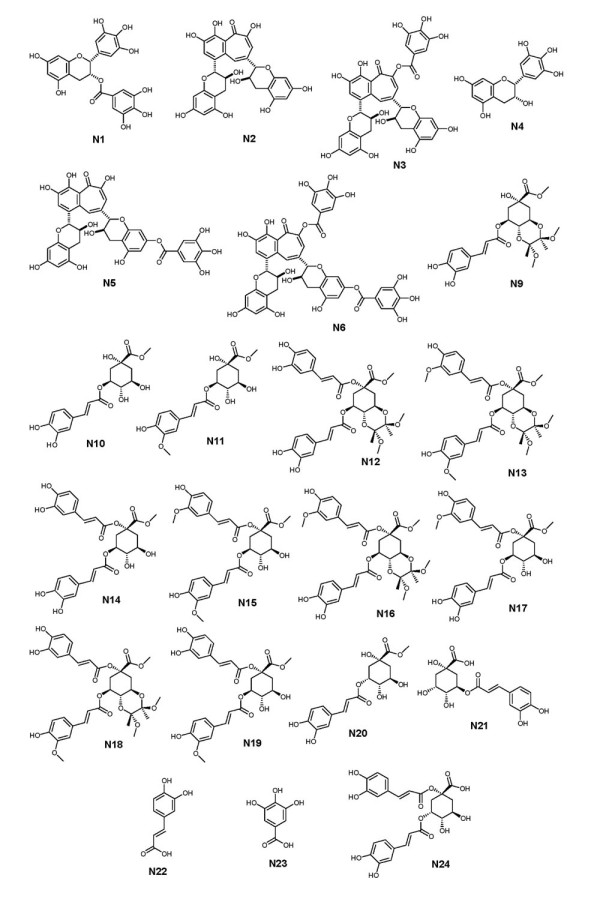
**Structures of the compounds tested for Dnmt3a-C inhibition**.

For this study, we first selected EGCG **N1 **and (-)-epigallocatechin **N4 **(from green tea, also on occasions found in black tea at low concentrations) as reference compounds. Next we selected the four most common theaflavin derivatives: theaflavin **N2**, theaflavin-3-gallate **N5**, theaflavin 3'-gallate **N3 **and theaflavin 3, 3'-digallate **N6 **(see Figure 2) [33,34]. All four compounds are found in black tea infusions at concentrations of around 100 mM, making up 2-3% of the total content of dry mass in typical black tea infusion. Theaflavins are structurally closely related the catechins being formal dimers of EGCG obtained through a two electron oxidation followed by C-C bond formation and a benzylic acid type rearrangement leading to the benztropololone core structure. Next to theaflavins we decided to screen as well two crude thearubigin fractions. We recently proposed that thearubigins contain several thousands polyhydroxylated theaflavin derivatives in equilibrium with their ortho-quinones. Theaflavins were obtained by extraction from black tea infusion followed by purification by preparative HPLC. The purity was assessed by LC-tandem MS. Thearubigins were obtained from black tea infusions using a protocol developed by Roberts [35].

For coffee polyphenols, we selected a range of naturally occurring and synthetic derivatives of chlorogenic acids. Chlorogenic acids (CGAs) are formally hydroxyl-cinnamate esters of quinic acid with a dietary intake of an estimated 2 g per human per day [36]. We recently reported a total of 70 different chlorogenic acids found in green coffee beans and selected some representative examples containing both caffeic acid and ferulic acid substituents [37-40]. Furthermore, we selected a range of epimers of CGAs produced from the original secondary plant metabolites by roasting of the coffee beans. Representative structures are shown in Figure 2. All CGA derivatives were obtained through chemical synthesis unless stated otherwise.

### Dnmt3a-C activity and inhibitors screening

**Figure 3:**
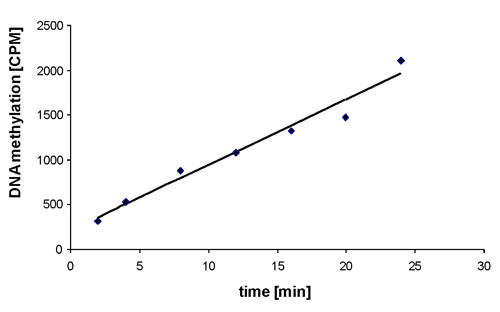
**Methyltransferase activity of the purified Dnmt3a-C**. Example of the methylation kinetics carried out with purified Dnmt3a-C. Initial slopes were determined by linear regression analysis of the initial linear parts of the reaction progress curves.

**Figure 4:**
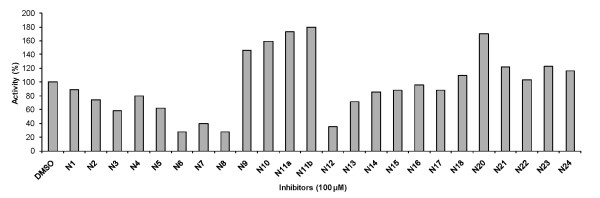
**Initial screening of the 24 compounds for inhibition of Dnmt3a-C**. Dnmt3a-C activity was determined in the presence of 100 μM compound. The control reaction was performed after adding a corresponding volume of DMSO.

The purified Dnmt3a-C was catalytically highly active (Figure 3). For an initial screening of the twenty four inhibitor candidates, Dnmt3a-C DNA methylation kinetics were carried out in the presence of 100 μM of compound. Rates of DNA methylation were derived by linear regression of the initial phase of the reaction progress curves. The reaction rates were compared with control reactions carried out after addition of a corresponding volume of DMSO to ensure identical reaction conditions, because DMSO had been shown before to influence the activity of Dnmt3a [41]. As shown in Figure 4, four of the compounds had a substantial inhibitory effect for the in vitro Dnmt3a-C activity (N6-N8 and N12). To determine IC_50 _values, DNA methylation kinetics were carried out in the presence of variable concentrations of the inhibitors, initial slopes derived and the activity profile analysed by fitting of the experimental data to the equation:

with: c_I_, concentration of the inhibitor; A(c_I_), activity in presence of inhibitor at concentration c; A_0_, activity in absence of inhibitor; BL, baseline.

**Figure 5:**
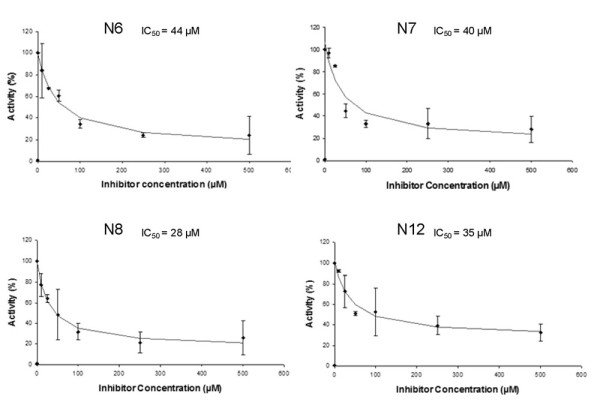
**Measurement of IC50 values for compounds N6, N7, N8 and N12**. For compounds N6, N7, N8 and N11 DNA methylation kinetics were carried out at different concentration of the compounds to determine the IC50 value. The error bars show the maximal deviations in repeated experiments. IC50 values are valid by ±30%.

As shown in Figure 5, the IC_50 _values for the compounds N6-N8 and N12 were all in the lower μM range.

## Discussion

Lee *et al *had showed that caffeic acid and chlorogenic acid inhibit the activity of M.SssI and Dnmt1 and decrease the methylation level at the RAR beta promoter gene in the breast cancer cell lines [12]. Furthermore, they have recently described the inhibition of human Dnmt1 by tea flavanoids such as EGCG, catechin and other flavanoids such as quercitin and myristin, observing K_I _values in the low micromolar range [11]. While Dnmt1 is considered a biological target involved in cancer development its close relative Dnmt3a, investigated in this study, has been linked to both cancer development and mental performance and health. Therefore, any inhibitory interaction between any of the screened dietary polyphenols and Dnmt3a might allow identification of compounds that have a positive effect on cancer prevention and improved mental performance.

### Black tea polyphenols

EGCG (N1) with a reported IC_50 _on Dnmt1 of 0.21 μM and epigallocatechin (N4) showed only weak inhibition of Dnmt3a. A slightly increased activity was observed for theaflavin, theaflavin-3-gallate (N3) and theaflavin 3'-gallate (N5) with the gallated derivatives showing a larger inhibitory effect. Theaflavin 3, 3'-digallate (N6) performed best in this series with a measured IC_50 _value of 44 μM. Similarly, the thearubigin fractions performed well in this test with IC_50 _values of 40 μM and 28 μM, respectively (molarity calculated by assuming an average molecular weight of 800 g/mol). It has to be noted that according to our knowledge this is the first time that a thearubigin fraction (consumed at a level of 1 Mt per annum) has been investigated in an enzyme assay and found to exhibit inhibitory activity. Previous work on thearubigins biological activity had focused on interference with signalling cascades in the anti-inflammatory response [42-45]. Due to the structural similarity of theaflavins and thearubigins (poly-hydroxy theaflavins), the inhibition of Dnmt3a does not come as a complete surprise.

To evaluate any possible biological significance of the IC_50 _values of Dnmt3a inhibition observed here, human pharmacokinetic data need to be consulted. Two published reports address the pharmacokinetic behaviours of theaflavins. Mulder and co-workers report theaflavin concentrations of 4.2 μg l^-1 ^in urine 2h after consumption of 1 cup of black tea containing 8.8 mg total theaflavins [46]. Henning reported a concentration of 2 nmol g^-1 ^tissue (if converted around 2 μM) of theaflavin in colon, small intestine, prostate and liver target tissue, with all further three theaflavins N3, N5 and N6 investigated here showing roughly 1 μM, half this value after consumption of one cup of black tea [47]. Although no plasma concentration values are available for theaflavin derivatives, it can be assumed that the plasma concentration is the same order of magnitude if not even higher when compared with concentrations in target tissues. As the average per capita consumption of black tea is around 550 ml or three cups per day, again a higher physiological concentration must be assumed.

From these data it becomes obvious that out of the compounds investigated theaflavin 3, 3'-digallate N6 is a compound showing reasonable bioavailability. These concentration estimate of 2 μM is only roughly by one order of magnitude smaller than the measured IC_50 _values. Assuming consumption of a black tea beverage rich in theaflavins (a maximum of 50 mg l^-1 ^has been determined) or repeated consumption of larger quantities of black tea the measured IC_50 _values for Dnmt3a inhibition, therefore, may have biological significance and inhibition of this enzyme can be expected under physiological conditions after black tea consumption. No data are available on thearubigin pharmacokinetics but since a typical cup of tea contains 60-70% of its dry mass of this mixture of compounds biological significance can as well be assumed.

Two pieces of further work published recently touch on the problem discussed here and are worth highlighting. Firstly, work by Vauzour et al. showed that dietary polyphenols from berries of similar polarity and structure compared to the polyphenols studied here, are able to cross the blood brain barrier [48], therefore suggesting that brain target tissue could be reached by the compounds under investigation. Secondly, recent work by Müller-Harvey et al. reports an accumulation of tea polyphenols in cell nuclei [49], suggesting that not only target tissue but target cell organelles, in which Dnmt3a methylates DNA can indeed be reached by the compounds under investigation.

### Coffee polyphenols

Out of the twelve chlorogenic acid derivatives screened, seven showed a minor inhibitory effect on Dnmt3a with one compound 1,3-dicaffeoyl-muco-quinic acid diacetal (N12) showing a good IC_50 _value of 35 μM. Since compound N12 is a synthetic derivative, not present in the human diet, this finding has no direct dietary significance. However, the activity of compound N12 clearly indicates that chlorogenic acid derivatives have the potential to inhibit Dnmt3a and this derivative might serve as a lead compound to screen and identify further dietary compound possessing this interesting biological activity.

Interestingly, all compounds showing inhibitory effects are diacyl quinic acids, whereas monoacyl quinic acids showed no effect at all. As a general trend caffeoyl derivatives seem to be more active if compared to feruloyl derivatives and a 1,3-diacyl regiochemistry appears to be favourable. Similarly gallic acid and caffeic acid had no inhibitory effect at all in contrast to the values reported by Lee & Zhu for Dnmt1 inhibition [12]. Despite the structural similarity of these two enzymes a predictive design of inhibitors targeting both classes of enzymes does not seem possible, which can be turned into an advantage considering that the compounds investigated by us and by Lee show remarkable selectivity for either Dnmt1 or Dnmt3a.

## Conclusions

We have shown that the black tea polyphenols, in particular theaflavin 3, 3'-digallate N6 and thearubigin fraction inhibit Dnmt3a with a physiologically and nutritionally relevant IC_50 _value and therefore identified a novel biological target that is able to rationalize both anti-carcinogenic activity and mental health and performance related activity of black tea.

## Methods

### Expression and purification of Dnmt3a-C

The mouse Dnmt3a C-terminal domain was expressed and purified as described [29,30]. The purity of protein was determined on 12% SDS-PAGE gel stained with colloidal Coomassie Blue (Figure 1). Protein concentration was determined from the absorbance at 280 nm using an extinction coefficient of 39290 M^-1 ^cm^-1^.

### DNA methyltransferase activity assay

Kinetics of Dnmt3a-C was analyzed by using a Biotin-Avidin methylation kinetics assay basically as described [50] using a biotinylated oligonucleotide substrate and [methyl-3H]AdoMet.

FP3 5'-TTGCACTCTCCTCCCGGAAGTCCCAGCTTC-3' FP3-Bt 5'-Bt-GAAGCTGGGACTTCCGGGAGGAGAGTGCAA-3'; The oligonucleotides were annealed by heating to 86°C for some minutes and slowly cooling down to ambient temperature. The methylation reactions were carried out in methylation buffer [20 mM HEPES pH 7.2, 1 mM EDTA, 50 mM KCl, 25 mg/ml bovine serum albumin (BSA)] at 37°C, using 1 μM substrate DNA, 0.76 μM AdoMet and 2.5 μM Dnmt3a-C. After the methylation reaction, the oligonucleotides were immobilized at various time points on an avidin-coated microplate. The incorporation of [3H] into the DNA was quenched by addition of an excess of unlabeled AdoMet to the binding buffer. Subsequently, unreacted AdoMet was removed by washing five times with PBST containing 0.5 M NaCl. The immobilized DNA was digested with a non-specific endonuclease to release the radioactivity from the microplate. After digestion, 120 μl of the reaction mixture were transferred to a fresh microplate and 160 μl of Microscint-PS scintillation fluid (Perkin Elmer) was added to each well. Finally, the amount of methyl groups transferred to the DNA and the solution obtained after nucleolytic digestion was quantified by using the TopCount NXT liquid scintillation counter. To determine the initial slope, the data were fitted by linear regression of the initial part of the reaction progress curves.

All the inhibitors were prepared in the DMSO at 5 mM stock. For the screening purpose 100 μM concentrations of the inhibitors were used in the reaction mixture. To detemine the apparent IC50 value for the potential inhibitors, different concentration of the inhibitors were used in the reaction mixture (10 μM, 25 μM, 50 μM, 100 μM, 250 μM, 500 μM). The different concentrations of the inhibitors were incubated with Dnmt3a protein for 10 min at room temp. The reaction was started by adding substrate and cofactor and further incubated at 37°C for another 10 min then the reaction was stopped by adding excess unlabelled AdoMet. The DMSO was used as control in each experimental setup to exclude the possible inhibition effect from the DMSO itself. All the inhibitor kinetics was done at duplicate and standard error was calculated for the two experimental values.

### Isolation and synthesis of inhibitors

EGCG N1 and (-)-epigallocatechin (N4), theaflavin (N2), theaflavin-3-gallate (N3), theaflavin 3'-gallate (N5) and theaflavin 3, 3'-digallate (N6) were from black tea obtained using published procedures (Figure 2) [33,51]. Thearubigin fractions (N7 and N8) were obtained from black tea and characterised using published procedures [33,51]. All chlorogenic acid derivatives were obtained by synthesis using published procedures (Figure 2) [52].

## Authors' contributions

AR, ZT and RJ conducted and analyzed the experiments. NK and AJ participated in the design of the study and in data analysis and interpretation and drafted the manuscript. All authors read and approved the final manuscript.
